# An inverse metabolic engineering approach for the design of an improved host platform for over-expression of recombinant proteins in *Escherichia coli*

**DOI:** 10.1186/1475-2859-11-93

**Published:** 2012-07-03

**Authors:** Chaitali Ghosh, Rashmi Gupta, Krishna Jyoti Mukherjee

**Affiliations:** 1 School of Biotechnology, Jawaharlal Nehru University, New Delhi, India

**Keywords:** Recombinant protein, Inverse metabolic engineering, *Escherichia coli*, Improved host platform

## Abstract

**Background:**

A useful goal for metabolic engineering would be to generate non-growing but metabolically active quiescent cells which would divert the metabolic fluxes towards product formation rather than growth. However, for products like recombinant proteins, which are intricately coupled to the growth process it is difficult to identify the genes that need to be knocked-out/knocked-in to get this desired phenotype. To circumvent this we adopted an inverse metabolic engineering strategy which would screen for the desired phenotype and thus help in the identification of genetic targets which need to be modified to get overproducers of recombinant protein. Such quiescent cells would obviate the need for high cell density cultures and increase the operational life span of bioprocesses.

**Results:**

A novel strategy for generating a library, consisting of randomly down regulated metabolic pathways in *E. coli* was designed by cloning small genomic DNA fragments in expression vectors. Some of these DNA fragments got inserted in the reverse orientation thereby generating anti-sense RNA upon induction. These anti-sense fragments would hybridize to the sense mRNA of specific genes leading to gene ‘silencing’. This library was first screened for slow growth phenotype and subsequently for enhanced over-expression ability. Using Green Fluorescent Protein (GFP) as a reporter protein on second plasmid, we were able to identify metabolic blocks which led to significant increase in expression levels. Thus down-regulating the *ribB* gene (3, 4 dihydroxy-2-butanone-4-phosphate synthase) led to a 7 fold increase in specific product yields while down regulating the gene *kdpD* (histidine kinase) led to 3.2 fold increase in specific yields.

**Conclusion:**

We have designed a high throughput screening approach which is a useful tool in the repertoire of reverse metabolic engineering strategies for the generation of improved hosts for recombinant protein expression.

## Background

Improvement of metabolic phenotype through directed genetic modifications is the main goal of metabolic engineering [1-4]. The classical approach of metabolic engineering requires a detailed knowledge of enzyme kinetics, the system network, and intermediate pools involved, and on this basis, a genetic manipulation is proposed for some presumed benefits. Recent progress in molecular genetics methods makes it possible to knockout or over-express targeted genes in most microorganisms [5-9]. A key question in metabolic engineering is how to identify gene targets that have direct or indirect impact on a particular phenotype of interest [10].

For the over-production of metabolites the issue is comparatively straight forward since only the regulatory blocks and bottlenecks in the specific pathway involved in product synthesis need to be removed. Secondly, the supply of precursor metabolites to the pathway need to be enhanced thereby improving the metabolic flux in the pathways. However, for products like recombinant proteins there is a vast range of precursors along with the transcriptional/translational mechanism all of which are coupled to the growth process. Many metabolic engineering approaches have therefore attempted to enhance both growth and recombinant protein production in *E. coli*[11,12]*.* One approach has been to supplement the genes which get down-regulated due to the stress associated with recombinant protein expression [13]. Similarly key factors which has impact on growth and expression yield, like glycolysis and Tri Carboxylic Acid (TCA) cycle enzymes, ATPase’s, RNA polymerases etc. have been co-expressed [14-16].

A novel alternative strategy to design overproducers of the recombinant product would be to make these precursors available specifically for product rather than biomass formation. We therefore decided to design a strategy to screen for metabolically active but non-growing quiescent cells which have been earlier shown to be more effective for recombinant protein production [17].

The concept of inverse metabolic engineering involves first to identify the desired phenotype, then to determine environmental or genetic conditions that confer this phenotype, and finally to alter the phenotype of the selected host by genetic manipulation [18,19]. In this study, we adopted this approach where an *E. coli* genomic library was screened to obtain quiescent cells which might be better producers of recombinant proteins. For this, we randomly and partially down-regulated the biosynthetic pathways in the cell, in order to identify those blockages which help in diverting the metabolic flux away from growth. Cells with slow growth phenotype or growth stoppage were then screened for enhanced protein expression capability. An additional advantage of non-growing, quiescent cells is that they can increase the operational life span of bio-processes and improve process economics by decoupling product formation from cell growth [20].

In this study an *E. coli* genomic library was prepared in a pRSET A vector having a strong promoter as well as in pBAD33 having a comparatively weaker promoter because even a small down regulation of some genes can have significant effects. Screening of the genomic library by different approaches (slow growth or enhanced GFP fluorescence) led to the identification of clones with different down-regulated genes which blocked growth and simultaneously improved recombinant protein expression.

## Results and discussion

### Identification of metabolic pathways which lead to 'no growth’ or slow-growth phenotype through genomic library screening

We constructed a library consisting of a set of clones with metabolic pathways which were ‘knocked-down’ rather than ‘knocked-out’, allowing us to include the role of essential genes. In our study the strategy adopted was to clone small fragments of genomic DNA (~ 200–800 bases), the idea being that some of these DNA fragments would get inserted in an opposite orientation (compared to the coding strand) in the expression vector. On induction, the RNA produced would be complimentary to the mRNA (of a functional protein) which would then hybridize leading to (partial) silencing of the gene. In eukaryotic systems an ideal antisense could be as short as a 22–25 bp fragment (as then a dicer molecule can act, leading to RNAi). But in prokaryotic systems, RNAi is absent and not many studies have been done on the antisense mechanism. So by choosing a 200–800 bp fragment we attempted to ensure that at least partial gene silencing would take place.

The concept of inverse metabolic engineering was used to screen this library for the desired ‘no-growth’ phenotype and then to identify the genetic factors that confer this phenotype. We were interested in a ‘no-growth’ or ‘slow-growth’ phenotype where the metabolic activity was undiminished; the rationale being such clones would divert the metabolic flux away from biomass formation and towards product synthesis.

### Construction of the antisense library in pRSET A vector

Genomic DNA fragments of sizes ranging from 200–800 bp (as we required only a part of the gene which would act in an antisense fashion to block transcription) were isolated after an optimized partial digestion of the *E. coli* genomic DNA and subsequently ligated into a high copy number vector (pRSET A) with a strong T7 promoter. The constructed shotgun library in pRSET A was transformed into *E. coli* BL21 pLysS strain.

**Figure 1:**
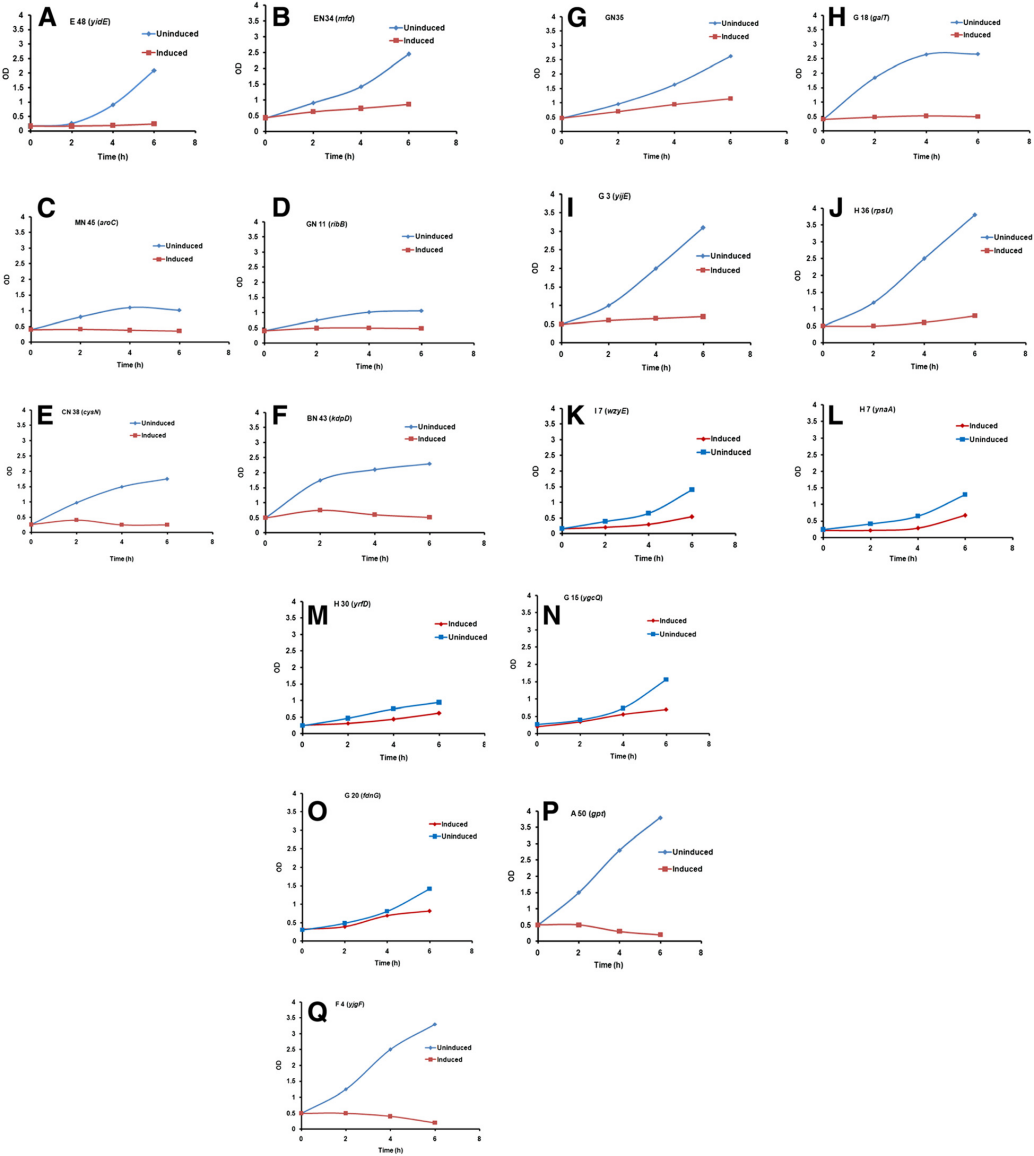
**Growth profiles of the clones in LB media under T7 promoter of pRSET A, in the host BL21 pLysS.(A)** E48 (*yidE*); **(B)** EN34 (*mfd*); **(C)** MN45 (*aroC*); **(D)** GN11 (*ribB*); **(E)** CN38 (*cysN*); and **(F)** BN43 (*kdpD*) **(G)** GN35; **(H)** G18 (*galT*); **(I)** G3 (*yijE*); **(J)** H36 (*rpsU*); **(K)** I7 (*wzyE*); and **(L)** H7 (*ynaA*) **(M)** H30 (*yrfD*); **(N)** G15 (*ygcQ*); **(O)** G20 (*fdnG*); **(P)** A50 (*gpt*) and; and **(Q)** F4 (*yjgF*) under (♦) uninduced; and (▪) IPTG induced condition respectively.

A two step screening strategy was used. In the first step, more than 8000 transformants were screened. Preliminary screening was done on plates. Each transformant was replica plated and only those clones were selected which upon Isopropyl β-D-1-thiogalactopyranoside (IPTG) induction grew very slowly on plates. After first screening, 728 clones were isolated and a second round of screening was done on LB agar plates. Finally 70 clones were picked and the metabolic activity of these slow growing transformants were checked in shake flask cultures containing M9 media (supplemented with glucose), by looking at rates of decline of glucose in the medium, post IPTG induction. Our aim was to identify clones with negligible growth rate but unimpaired metabolic activity. 17 transformants were finally selected with this desirable phenotype where it was expected that the metabolic flux would possibly get diverted towards recombinant protein expression instead of growth. Shake flask studies of all these 17 clones were done in different media (LB, TB & M9) where a similar retardation in growth was observed. As is clear from Figure 1 significant growth retardation was observed post induction compared to the uninduced cultures.

These clones were sequenced and used to do a BLAST search against the *E. coli* genome database. The lists of identified genes are given in Table 1. The details of the genes were collected from the PEC (Profiling of E. coli Chromosome) and Ecocyc database.

**Table 1:** List of transcripts whose blockage leads to growth stoppage

**Transcript name**	**Gene name**	**Size (bp)**	**Known or putative function of the gene product**	**Orientation**	**Essential/ Non essential**
GN35 (T7)	*-*	221	Ketoacid-binding protein	Reverse	Non-essential
CN38 (T7)	*cysN*	567	Adenosine 5’ phosphosulphate kinase sulfate adenylyltransferase subunit 1	Same direction	Non-essential
BN43 (T7)	*kdpD*	568	Sensory histidine kinase	Reverse	Non-essential
GN11 (T7)	*ribB*	403	3,4 dihydroxy-2-butanone-4-phosphate synthase	Reverse	Non-essential
EN34 (T7)	*mfd*	587	Transcription repair coupling factor	Same direction	Non-essential
MN45 (T7)	*aroC*	190	Chorismate synthase	Reverse	Non-essential
G3 (T7)	*yijE*	379	Predicted permease	Reverse	Non-essential
A50 (T7)	*gpt*	141	Xanthine-guanine phosphoribosyl transferase	Reverse	Non-essential
F4 (T7)	*yjgF*	122	Ketoacid binding protein	Reverse	Non-essential
H36 (T7)	*rpsU*	422	Acid resistant protein	Same direction	Non-essential
E48 (T7)	*yidE*	350	Predicted transporter	Reverse	Non-essential
G15 (T7)	*ygcQ*		Predicted electron transfer flavoprotein, NAD/FAD binding	Same direction	Non-essential
G18 (T7)	*galT*	375	Galactose −1 phosphate uridylyltransferase	Reverse	Non-essential
G20 (T7)	*fdnG*	720	Formate dehydrogenase- N,alpha subunit,nitrate inducible	Reverse	Non-essential
H7 (T7)	*ynaA*	876	Conserved hypothetical protein: Rac prophage	Reverse	Non-essential
H30 (T7)	*yrfD*	769		Reverse	Non-essential
I7 (T7)	*wzyE*	770	Predicted Wzy protein involved in ECA polysacharide	Same direction	Non-essential
L27 (ara)	*-*	639	Conserved hypothetical protein	Reverse	Non-essential
A17 (ara)	*bioF*	414	Predicted methyltransferase, enzyme of biotin synthesis	Reverse	Non-essential
A29 (ara)	*kdpF*	289	2-octaprenylphenol hydroxylase enzyme	Reverse	Non-essential
F50 (ara)	*-*	313	iro p300	Reverse	Non-essential

These 17 clones were co-transformed with a second plasmid pBAD33-GFP, where the GFP gene was cloned under the arabinose promoter and had a compatible p15 ‘ori’ with pUC ‘ori’ of pRSET A. This GFP reporter gene was used to check whether the antisense expression (while blocking growth) also helped in the overexpression of recombinant protein. All these experiments were done in LB media. For all the 17 clones one flask was induced only with 0.02% of arabinose (control), while the second test flask was co-induced with both IPTG and arabinose, to check the effect of the antisense transcript on GFP expression. A third flask for all the co-transformants was kept uninduced to observe the effect if any, of leaky expression (2^nd^ control). The library contained large number of random inserts in pRSET A (which did not show any affect) which essentially served as a negative control.

**Figure 2:**
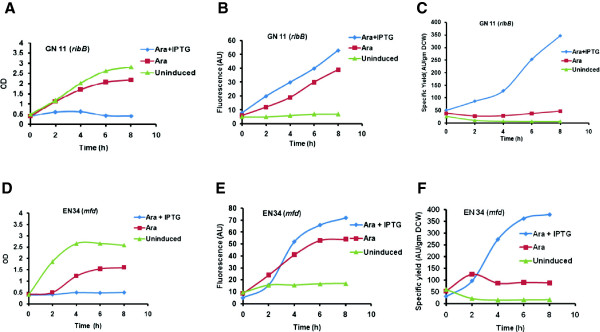
**Time profiles of growth, Fluorescence spectra and Specific product yields of GN11 (*ribB*) in A, B, and C and EN34 (*mfd*) in D, E, and F respectively in LB media.** (▴) uninduced; (▪) induced with arabinose; (♦) induced with arabinose and IPTG.

Figure 2 (A,B,C) shows the effect of co-expression of clone GN11 having the anti-sense of *ribB* gene (rib-3, 4 dihydroxy-2-butanone-4-phosphate synthase). In Figure 2B the uninduced culture had a basal level GFP expression of 5 to 7 AU while GFP expression in the *ara* induced control culture increased linearly from 6 to 39 AU in 8 hours post induction. In the test system the fluorescence of the co-induced culture overtook the control flask after 2 hours to give a final fluorescence of 53 AU demonstrating the positive effect of antisense induction on GFP expression. Since the final biomass concentrations obtained in the test culture was significantly lower, the specific product yield was much higher at 347 AU/g DCW, a 7 fold increase over the control culture (47 AU/g DCW) (Figure 2C).

Figure 2 (D,E,F) shows the co-expression of the clone EN34 coding for mutation frequency decline (*mfd*) protein. In Figure 2E the uninduced flask showed a basal level expression of 16.9 AU throughout the cultivation. The control flask showed a linear increase from 10 AU to 54 AU in 8 hours. However, while the expression was initially low in the co-induced culture, it overtook the control flask after 3 hours and gave a final GFP fluorescence of 72 AU. The specific product yield of arabinose induced GFP was found to be 88 AU/gm DCW in the 8^th^ hour post induction in the control culture whereas in the arabinose and IPTG induced culture it was calculated to be 378 AU/gm DCW which was 4 fold higher (Figure 2F).

Similar studies were conducted with the rest of the 15 clones. However, not all the clones gave such a dramatic increase in the specific product yield though we did observe a significant increase in specific product yield in some cases which are listed in Table 2*.*

**Table 2:** Specific product yield (AU/gm DCW) of various clones under three different conditions, uninduced, induced with arabinose and induced with arabinose and IPTG both (6 hrs post induction)

**a) Clones in pRSET A**			
**Clone name**	**Specific product yield (Ara + IPTG)**	**Specific product yield (Ara)**	**Specific product yield (Uninduced)**
EN34 (*mfd*)	362	91	14
G18 (*galT*)	76	227	18
GN35	89	244	9
GN11 (*ribB*)	347	47	8
MN45 (*aroC*)	67	199	25
BN43 (*kdpD*)	256	67	10
b) Clones in pBAD 33			
A17 (*bioF*)	125	181	9
A29 (*kdpF*)	681	79	5
F50	225	207	10
L27	175	175	13

### Construction of the antisense library in pBAD33

In the previous screening strategy we over-expressed the transcripts under the strong T7 promoter, which has certain disadvantages. Firstly, IPTG itself is partially toxic to cells and also the expression of T7 RNA polymerase leads to growth retardation [21,22]. This would mask the true growth retardation due to the anti-sense effect of the transcripts. Secondly, since the anti-sense works at the RNA level we should use only a transcriptional vector and not an expression vector to rule out the possibility that the toxicity of a randomly expressed polypeptide is responsible for growth retardation. Thirdly, hyper expression of the anti-sense RNA seems to be a clumsy approach to block and/or down-regulate gene expression (even though this was primarily a screening strategy for identifying potential targets for manipulation). We therefore chose a much better regulated transcriptional vector for the construction of a second library which would identify targets where even a partial down-regulation would lead to diversion of metabolic flux towards product synthesis.

The genomic library was prepared under the *ara* promoter in pBAD33 (a transcriptional vector), which is a tightly regulated promoter. Moreover as we were not constrained to use the BL21 (DE3) strain we chose *E. coli* DH5α since it has superior transformation efficiency and is much more stable strain for retaining plasmids. A dual plasmid system was used to directly screen for the desired phenotype. Competent DH5α cells pre-transformed with a plasmid pNER31 (having GFP under the *lac* promoter) was used for co-transformation with the genomic library. This allowed us to directly screen for the over-expressing phenotype. Upon co-induction of the transcripts, we screened for colonies exhibiting intense green color of GFP (under UV) compared to the control colonies in the plate. More than 30,000 transformants of the library was screened. Finally 4 clones (Table 1), exhibiting higher GFP expression was selected for further studies. Shake flask studies on these clones were done in different media LB, TB, M9 along with appropriate controls.

**Figure 3:**
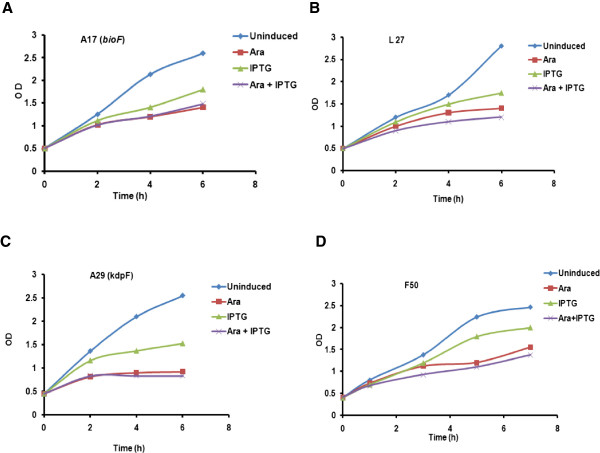
**Growth profiles of the clones in LB media under ara promoter of pBAD33, in the host DH5α.(A)** A17 (*bioF*); **(B)** L27; **(C)** A29 (*kdpF*); **(D)** F50 under (♦) uninduced; (▪) induced with arabinose; (▴) induced with IPTG and (**×**) induced with arabinose and IPTG condition respectively.

We first needed to factor out the effect of GFP expression on growth retardation by comparing the growth of the uninduced culture with the IPTG induced culture. A very slight decline in growth was observed post induction in LB media which is typical of GFP induced cultures. However when the second, anti-sense gene carrying plasmid, was induced by arabinose a significant drop in growth was observed. The growth profile of the co-induced culture was compared with the control culture where only GFP was induced. We observed that the growth declined sharply in all the co-induced cultures (Figure 3).

**Figure 4:**
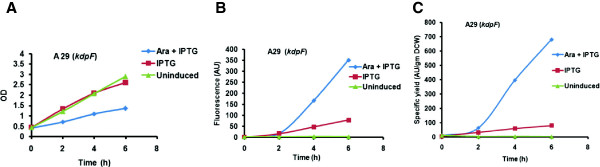
**Time profiles of growth, Fluorescence spectra and Specific product yields of A29 (*kdpF*) in A, B, and C respectively in LB media.** (▴) uninduced; (▪) induced with IPTG; (♦) induced with arabinose and IPTG.

Comparing the product profile of *kdpF* (2-octaprenylphenol hydroxylase enzyme) antisense expressing clone A29, we observed that, the specific product yield of the co-induced culture was 8.4 fold higher than the control culture which was induced with IPTG only (Figure 4). Co-expression studies were also done with rest of the 3 clones (data not shown).

One of the open questions in the quest for improved host platforms is how targets are to be identified which confer the desired phenotype, given the lack of a comprehensive kinetic model for *E. coli* and the absence of detailed information for the regulatory mechanism, which operate inside the cells. There are therefore several approaches for the modifications of the *E. coli* host to achieve the desired goal [23-25]. In our case the design strategy was essentially a high throughput screen which helped us select better producers. It is interesting to note that all the selected genes were non-essential and also not directly linked to either growth or product formation.

For example, the selected clone GN11 contained an insert of 403 bp of the *ribB* gene (3, 4 dihydroxy-2-butanone-4-phosphate synthase) in the reverse direction. *ribB* serves as the biosynthetic precursor for the xylene ring of riboflavin [26,27]. It is reported that *ribB* mutant *E. coli* leads to slow growth but remains metabolically active which corroborates our studies [28]. However the discovery that blocking the gene *ribB* can help divert the metabolic flux towards recombinant protein production was both non obvious and counter intuitive. Similarly, clone BN43 coded for a protein KdpD (sensory histidine kinase) which is expressed during osmotic shock and is otherwise nonessential. Clearly in this case there is no direct link to the gene function and the observed channelization of metabolic flux towards recombinant protein production. In the case of clone MN45 which contained part of the antisense *aroC* (chorismate synthase gene, which catalyzes the formation of chorismate in aromatic amino acid biosynthesis) its blockage should ideally lead to a decrease in the biosynthetic pathways involved in growth. However, an exact estimate of the growth inhibition is difficult given that complex nitrogen sources are present in the medium which can supply the growing cell with the desired amino acids. The fact that blocking chorismate synthase led to a complete growth stoppage was interesting and points to the criticality of even single metabolic reactions in growth. All these results underscore the fact that there is no direct link at the pathway level between gene function and observed phenotype which could possibly be the result of a complex regulatory response within the cell.

## Conclusion

In a novel approach towards the design of an ideal host platform for the over-expression of recombinant proteins, two different high throughput screening strategies were designed to identify genes whose down-regulation would lead to a slow growth phenotype and also possibly divert the metabolic flux towards over-expression of recombinant proteins. The leads obtained did not rely on a *priori* knowledge of the regulated and interconnected nature of the *E. coli’s* metabolic network. At a practical level we were able to obtain very useful targets for gene knock-out/knock-down which would enable the design of better hosts for protein expression. It would be interesting to study the combined and synergistic effect of these identified blocks. At a more fundamental level a detailed analysis of the phenotypic affects of these metabolic blocks can lead to a better understanding of the regulatory mechanisms in the *E. coli* network.

## Materials and methods

### Growth media and antibiotics

Media and bulk chemicals were purchased from local manufacturers, Himedia, Qualigens, Difco and MERCK. Media used were Luria Broth [LB] (bactotryptone 10 g/l, yeast extract 24 g/l and NaCl 15 g/l), Terrific Broth [TB] (YE 24 g/l, tryptone 12 g/l, glucose 0.5% in which 2.31 g/l KH_2_PO_4_ and 12.54 g/l K_2_HPO_4_ were added after autoclaving separately), and minimal media [M9] (NaCl 0.5 g/l, NH_4_Cl 1 g/l, K_2_HPO_4_ 3 g/l, 1 M CaCl_2_ 0.1 ml/l, 1 M MgSO_4_ 2 ml/l and 20% glucose 10 ml/l). Antibiotics used were ampicillin 100 μg/ml (1X), kanamycin 50 μg/ml (1X) and chloramphenicol 30 μg/ml (1X).

### Bacterial strains and plasmids

The T7 based expression vector pRSET A (ampicillin, Invitrogen) which had pUC ‘ori’, was used in these studies. pBAD33 (chloramphenicol, Beckwith) [29] having p15 ‘ori’ with *ara* promoter and compatible with pRSET A, was used for expression of GFP gene (used as a reporter protein) and the construction of second library. The plasmids were maintained and expressed in *E. coli* DH5α cells. *E. coli* BL21 (DE3) from stratagene (USA) and *E. coli* BL21 (DE3) pLysS were used for expression studies. Strain JM109 (Promega, USA) was used for the genomic library preparation. pNER31 (ampicillin) having GFP gene under ‘*lac*’ promoter and induced by IPTG, was gifted by Summers, Cambridge, UK and pET14b-GFP (ampicillin) where GFP gene was cloned under T7 promoter, was gifted by J. Singh, ICGEB, India.

### Construction of genomic library in vectors pRSET A and pBAD33

Genomic DNA was isolated from JM109 by the Hexadecyltrimethylammonium bromide (CTAB) method. This was partially digested with either *Sau*IIIA or *Nla*III both four base cutters. The expression vectors pRSET A and pBAD33 were digested with *Bgl*II and *Sph*I which generate compatible cohesive ends with *Sau*IIIA and *Nla*III respectively. These linearised vector fragments were Calf Intestinal Alkaline Phosphatase (CIAP) treated to prevent self ligation and ligated with the digested and gel eluted genomic fragments of size ranging from 200 to 800 bp. The ligation mixture was used to transform electrocompetent BL21(DE3) pLysS and DH5α cells respectively and plates were incubated at 37°C overnight. Approximately 8000 and 30,000 colonies were obtained in each library respectively. The quality of the library that was constructed was checked by picking a few colonies and checking for the insert size by colony PCR.

### Screening of the library by two different approaches

A preliminary screening of the pRSET A vector library was done on LB agar plates. Replica plating was done onto two different plates. One plate containing IPTG was considered as induced and the second plate without IPTG was considered as control. Slow growing colonies upon induction were identified and there growth profile was determined by performing shake flask studies in different media viz. LB, TB and M9.

For the second library under pBAD33, a co-transformation protocol was followed where electrocompetent cells of DH5α already containing the pNER31 plasmid (with GFP) was prepared by glycerol wash method and electroporated with the ligation mixture containing the genomic library. Primary screening was done on LB agar plates by induction of both the plasmids with IPTG (1 mM final concentration) and arabinose (0.02%, final concentration). Colonies with enhanced fluorescence were picked, and the genomic fragments cloned in the second plasmid (containing the library) which were responsible for enhanced fluorescence, was determined by sequencing. The growth and product profiles were determined for the selected clones by shake flask studies which were done in M9, TB and LB media.

Selected clones which showed enhanced fluorescence upon induction were grown overnight with shaking at 37°C in 3 ml LB tubes. Secondary inoculation was done by adding 100 μl of overnight grown culture in 10 ml of LB, TB and M9 medium in 100 ml flasks. After 1.5 hours when the OD_600_ of 0.3-0.5 was attained, cultures were induced by adding 1 mM IPTG (final concentration). After induction, the OD_600_ was monitored at regular intervals.

For measuring the metabolic activity of non growing cells in minimal media, 200 μl of samples were taken at regular intervals post induction. These samples were centrifuged at 13,200 rpm for 2 minutes and the supernatant was stored at 4°C. Glucose levels were estimated by the 3, 5-Dinitrosalicylic acid (DNSA) method.

The expression of GFP was monitored by Varian (Carry Eclipse) Fluorescence Spectrophotometer with an excitation wavelength of 488 nm and emission wavelength of 514 nm.

The specific yield was calculated using the formula Fluorescence/OD = AU/gm DCW (the dry cell weight factor is 0.3 gm/L/1OD600).

## Abbreviations

GFP: Green fluorescent protein; CTAB: Hexadecyltrimethylammonium bromide; DNSA: 3, 5-Dinitrosalicylic acid; CIAP: Calf Intestinal alkaline phosphatase; IPTG: Isopropyl β-D-1-thiogalactopyranoside.

## Competing interests

The authors declare that they have no competing interests.

## Authors’ contributions

CG and KJM designed the experiments and wrote the manuscript. CG performed all the experiments. RG performed few experiments. All authors read and approved the final manuscript.
